# Concurrent Caudal Occipital Malformation Syndrome-Associated Syringomyelia and Presumptive Meningoencephalomyelitis of Unknown Origin Managed with Combined Surgical Decompression and Immunosuppressive Therapy in a Dog

**DOI:** 10.3390/vetsci13080736

**Published:** 2026-07-24

**Authors:** Sung Su Park

**Affiliations:** IU Animal Medical Center, Seongnam-si 13646, Republic of Korea; slugger98@hanmail.net

**Keywords:** caudal occipital malformation syndrome, syringomyelia, meningoencephalomyelitis of unknown origin, magnetic resonance imaging, foramen magnum decompression, leflunomide, mycophenolate mofetil, dog

## Abstract

A 2-year-old spayed female Bichon Frise presented with progressive neurologic deterioration despite 12 months of medical management for caudal occipital malformation syndrome (CM)-associated syringomyelia (SM) and ventriculomegaly. Repeat magnetic resonance imaging (MRI) performed prior to surgical intervention demonstrated persistent CM-associated structural abnormalities together with multifocal intra-axial lesions involving the bilateral thalami, frontal white matter, and medulla that were considered most compatible with a presumptive inflammatory meningoencephalitic process, with MUO regarded as the leading differential diagnosis. Combined surgical decompression and immunosuppressive therapy using leflunomide and mycophenolate mofetil were subsequently initiated. Progressive clinical improvement followed by long-term neurological stability was observed during the approximately 2-year follow-up period. However, because both interventions were initiated concurrently, the relative contribution of each treatment to the observed clinical outcome could not be determined. No recurrence or progression of neurological signs was observed during the approximately 2-year follow-up period. This case highlights the importance of repeat MRI evaluation in neurologic patients exhibiting progressive deterioration despite previous treatment, even when an established structural diagnosis is present. Recognition of MRI findings suggestive of concurrent inflammatory disease may substantially influence diagnostic interpretation and therapeutic decision-making in selected patients.

## Simple Summary

A young Bichon Frise had persistent neck pain and worsening gait abnormalities despite prolonged medical treatment for a congenital abnormality at the junction between the skull and neck, accompanied by fluid-filled cavities within the spinal cord. Repeat magnetic resonance imaging performed before surgery showed not only the previously recognized structural abnormalities but also additional brain lesions suggestive of an inflammatory disorder that had not been recognized on the initial examination. The dog underwent decompressive surgery with reconstruction using a titanium mesh plate and simultaneously received long-term immunosuppressive treatment with leflunomide and mycophenolate mofetil. Neurologic function improved and remained clinically stable for approximately two years. Because cerebrospinal fluid analysis, tissue examination, and postoperative magnetic resonance imaging were not performed, the inflammatory diagnosis remained presumptive, and the respective contributions of surgery and medical treatment could not be determined. This case illustrates the value of repeating diagnostic imaging when neurologic signs continue to worsen despite treatment of a known structural disorder and emphasizes the need for cautious interpretation when structural and presumptive inflammatory neurologic diseases coexist.

## 1. Introduction

Caudal occipital malformation syndrome (CM), also referred to as Chiari-like malformation, is a developmental disorder of the caudal cranial fossa characterized by overcrowding within the caudal fossa, cerebellar compression, and abnormal cerebrospinal fluid (CSF) flow dynamics in small-breed dogs [1,2]. Progressive disruption of CSF circulation at the level of the foramen magnum may result in ventriculomegaly and syringomyelia (SM), which represent major secondary consequences of CM [1,2,3,4]. Affected dogs commonly develop cervical pain, phantom scratching, gait abnormalities, paresis, and multifocal neurologic dysfunction, although the severity of clinical signs does not always correlate with the magnitude of MRI abnormalities [2,4]. Although medical management using corticosteroids, gabapentin, pregabalin, proton pump inhibitors, or diuretics may temporarily alleviate clinical signs, progressive neurologic deterioration frequently occurs in dogs with persistent caudal fossa overcrowding and ongoing CSF flow disturbance, making surgical foramen magnum decompression (FMD), with or without cranioplasty, a widely accepted treatment option for dogs with clinically significant CM/SM, particularly those demonstrating progression despite medical management [2,3,4]. Recent surgical strategies have increasingly emphasized the importance of maintaining long-term decompression and reducing secondary scar-associated re-obstruction [5].

MUO represents a spectrum of presumptive immune-mediated inflammatory diseases of the canine central nervous system, including granulomatous meningoencephalomyelitis, necrotizing meningoencephalitis, and necrotizing leukoencephalitis, and predominantly affects small-breed dogs [6,7,8]. These disorders are typically characterized by progressive multifocal neurologic deficits and inflammatory intra-axial lesions on MRI [6,7,8,9]. Conventional treatment relies heavily on glucocorticoids, either alone or in combination with additional immunosuppressive agents [6,7,8]; however, increasing concern regarding long-term corticosteroid-associated adverse effects has prompted interest in glucocorticoid-sparing immunosuppressive protocols, and the author previously described apparent MRI-confirmed remission of presumptive MUO using leflunomide and mycophenolate mofetil (MMF) in a single patient [10]. However, evidence supporting glucocorticoid-sparing protocols in canine MUO remains limited, and further studies involving larger case numbers are required before broader conclusions can be drawn.

Progressive neurologic deterioration in dogs with CM/SM is generally attributed to persistent structural abnormalities and associated CSF flow disturbances alone. However, concurrent inflammatory intracranial disease may substantially complicate both diagnosis and treatment planning, as both CM/SM and MUO can present with overlapping clinical signs including cervical pain, gait abnormalities, paresis, and progressive neurologic decline. Furthermore, the potential relationship between chronic structural CSF abnormalities and concurrent inflammatory intracranial disease remains poorly understood. To the authors’ knowledge, no previous reports have described the combined management of these two conditions using FMD together with long-term immunosuppressive therapy in the same patient.

The present report describes the diagnostic reassessment, repeat MRI findings, treatment strategy, and long-term clinical outcome of a dog with CM-associated ventriculomegaly and syringomyelia in which repeat MRI demonstrated additional multifocal intra-axial lesions suggestive of a presumptive inflammatory meningoencephalitic process, with MUO regarded as the leading differential diagnosis. The patient was subsequently managed using FMD, C1 dorsal laminectomy, titanium mesh plate cranioplasty, and long-term immunosuppressive therapy consisting of leflunomide and MMF.

## 2. Case Description

A 2-year-old, 4.3 kg spayed female Bichon Frise was referred to IU Animal Medical Center for surgical evaluation of progressive neurologic deterioration. The dog had a previously established diagnosis of CM-associated SM and ventriculomegaly. At the time of referral, neurologic examination revealed cervical hyperesthesia, ambulatory tetraparesis with proprioceptive deficits in all four limbs, and reduced postural reactions. No cranial nerve deficits or seizure activity were identified.

**Figure 1:**
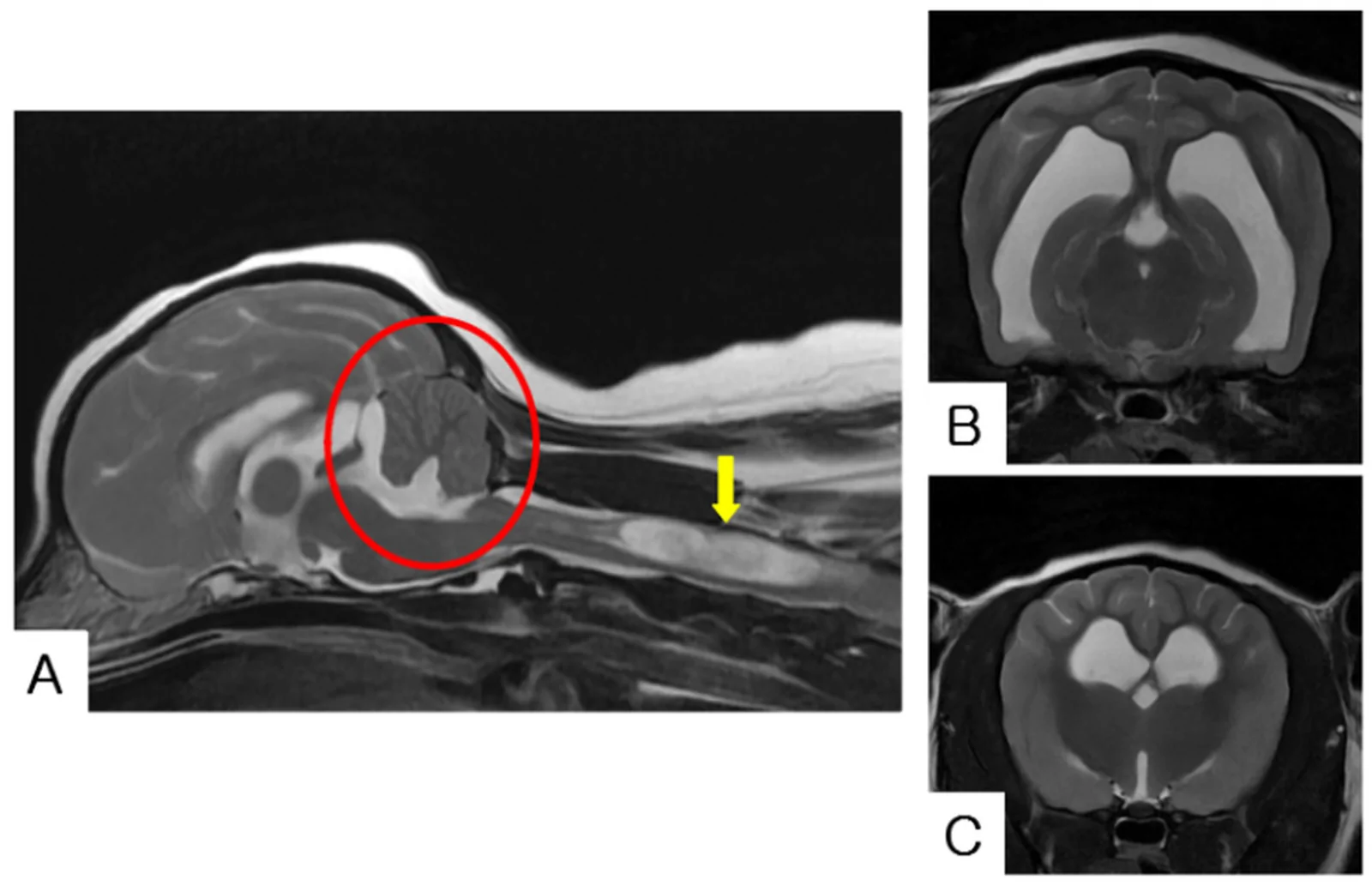
Magnetic resonance imaging (MRI) findings obtained at the time of the initial diagnosis, approximately 12 months prior to referral for surgical treatment. (**A**) Mid-sagittal T2-weighted image demonstrating caudal fossa overcrowding with mild cerebellar indentation/compression consistent with caudal occipital malformation syndrome (CM). Marked dilation of the third and fourth ventricles is evident, accompanied by syringomyelia involving the cervical spinal cord. Caudal fossa compression and dilation of the third and fourth ventricles are indicated by red circles, whereas the syringomyelia within the cervical spinal cord is indicated by yellow arrows. (**B**,**C**) Transverse T2-weighted images demonstrating marked ventriculomegaly, including symmetric dilation of the lateral ventricles and enlargement of the fourth ventricle. Mild periventricular T2 hyperintensity is additionally observed. Mild caudal fossa crowding is present.

Approximately 12 months prior to referral, the dog had initially presented to a referring veterinary hospital because of episodic cervical rigidity and cervical discomfort. Over the subsequent months, progressive worsening of neurologic signs was observed, including deterioration of gait and persistent cervical pain. MRI performed at that time demonstrated caudal fossa overcrowding with loss of normal occipital bone contour, mild cerebellar compression/indentation, marked dilation of the third and fourth ventricles, and cervical syringomyelia, findings compatible with CM/SM (Figure 1A–C). Cerebrospinal fluid (CSF) analysis was not performed at that time owing to concerns regarding altered intracranial pressure dynamics associated with the severe craniocervical structural abnormalities. Following the initial diagnosis at the referring hospital, medical management consisting of prednisolone (0.5 mg/kg, twice daily) and acetazolamide (5 mg/kg, twice daily) was initiated, resulting in transient improvement of clinical signs. However, despite continued medical therapy, cervical discomfort and gait abnormalities progressively worsened over the subsequent months. Approximately 12 months after the initial diagnosis, continued deterioration of neurologic signs prompted the referring veterinarian to recommend surgical decompression, and the patient was subsequently referred for surgical treatment.

**Figure 2:**
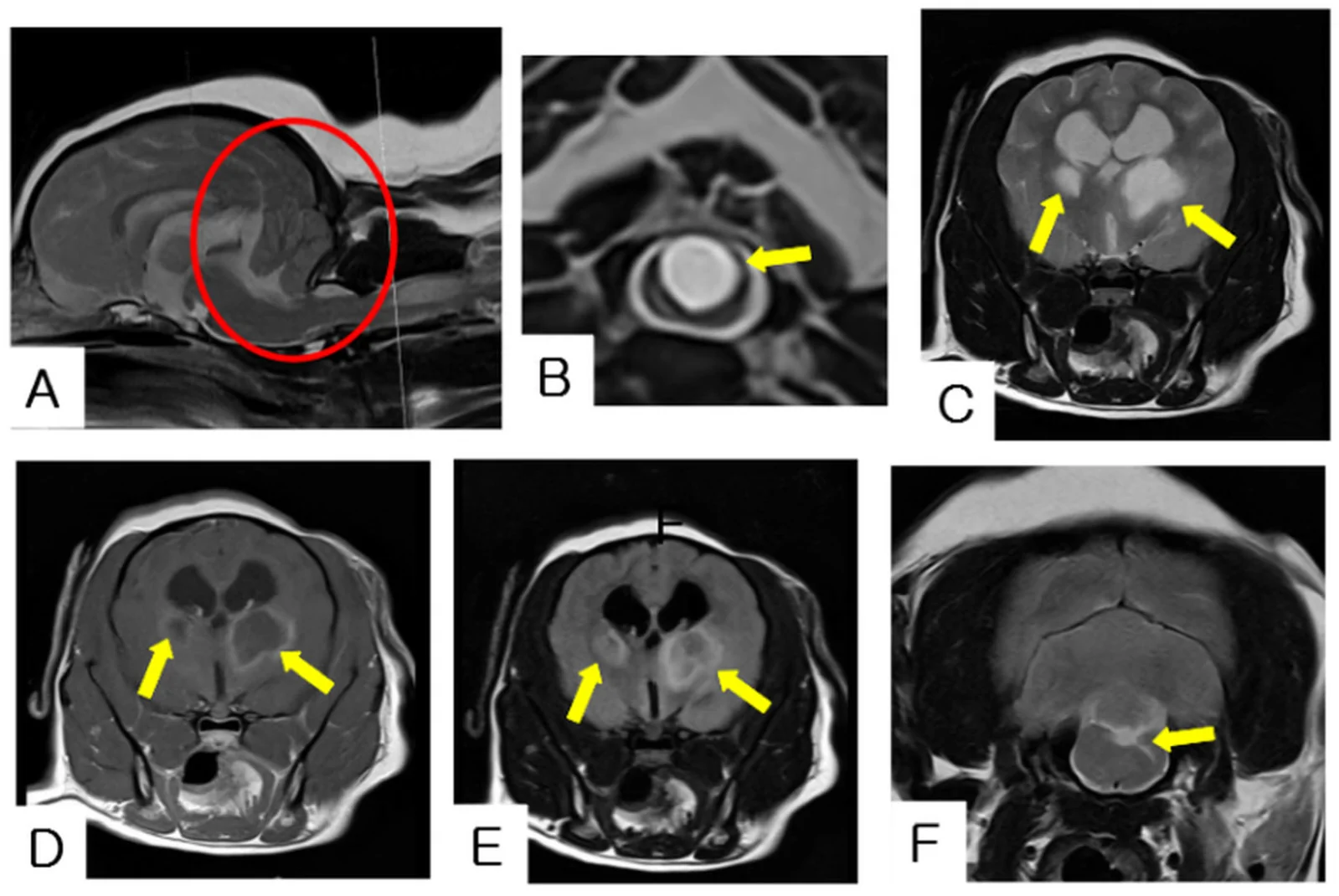
Repeat magnetic resonance imaging (MRI) findings obtained 12 months after the initial diagnosis following progressive neurologic deterioration despite medical management. (**A**) Mid-sagittal T2-weighted image demonstrating persistent caudal fossa overcrowding, ventriculomegaly, and syringomyelia identified at the time of repeat MRI examination (red circle). Persistent syringomyelia involving the cervical spinal cord is also identified. Direct comparison of the degree of cerebellar compression with the initial MRI study should be interpreted cautiously, as differences in head and cervical positioning between the two examinations may influence the apparent degree of caudal fossa crowding on sagittal images. (**B**) Transverse T2-weighted spinal cord image demonstrating syringomyelia characterized by a cavitary intramedullary T2 hyperintense lesion within the cervical spinal cord (yellow arrow). (**C**) Transverse T2-weighted image demonstrating bilateral thalamic hyperintense intra-axial lesions (yellow arrows). (**D**) Transverse post-contrast T1-weighted image demonstrating corresponding bilateral thalamic hypointense lesions with peripheral contrast enhancement (yellow arrows). (**E**) Transverse fluid-attenuated inversion recovery (FLAIR) image demonstrating bilateral thalamic hyperintense lesions (yellow arrows). Part of the left thalamic lesion demonstrates focal centrally isointense signal intensity on FLAIR imaging. (**F**) Transverse T2-weighted image demonstrating a focal hyperintense lesion within the left medullary region (yellow arrow).

Because a substantial interval had elapsed since the initial MRI examination and neurologic signs had continued to worsen despite medical management, repeat MRI evaluation was performed before surgical intervention and preoperative and follow-up CT examinations were performed. Repeat MRI demonstrated persistent CM-associated caudal fossa overcrowding together with persistent ventriculomegaly and SM (Figure 2A,B). Although the cerebellar indentation appeared subjectively more pronounced on the follow-up examination, objective assessment of structural progression could not be reliably determined because the two MRI studies were obtained at different institutions under different imaging conditions and head positioning. CSF analysis was not performed at this examination because surgical decompression was prioritized owing to concerns regarding altered intracranial pressure dynamics associated with the severe craniocervical structural abnormalities. In addition to the previously identified structural abnormalities, multifocal intra-axial lesions were identified involving the bilateral thalami and bilateral frontal white matter regions. These lesions were characterized by T2-weighted hyperintensity, post-contrast T1-weighted hypointensity with peripheral contrast enhancement, and fluid-attenuated inversion recovery (FLAIR) hyperintensity (Figure 2C–E), findings consistent with inflammatory parenchymal disease previously described in canine MUO [5,6,7,8,10]. Although these lesions were not identified on the initial MRI examination, it cannot be excluded that subtle inflammatory abnormalities may have been present but remained radiologically unapparent at that time. Part of the left thalamic lesion demonstrated centrally isointense-to-hypointense signal intensity on FLAIR images (Figure 2E). An additional focal T2 hyperintense lesion was identified within the left medullary region (Figure 2F). Collectively, these MRI findings were considered most compatible with an inflammatory meningoencephalitic process, with presumptive MUO regarded as the leading differential diagnosis. However, because cerebrospinal fluid analysis and histopathologic confirmation were unavailable, infectious, vascular, and neoplastic diseases could not be definitively excluded. Based on the overall clinicoradiological assessment, presumptive MUO was considered the most likely clinical diagnosis among the differential diagnoses. Nevertheless, definitive etiologic differentiation was not possible without cerebrospinal fluid analysis and histopathologic confirmation.

**Figure 3:**
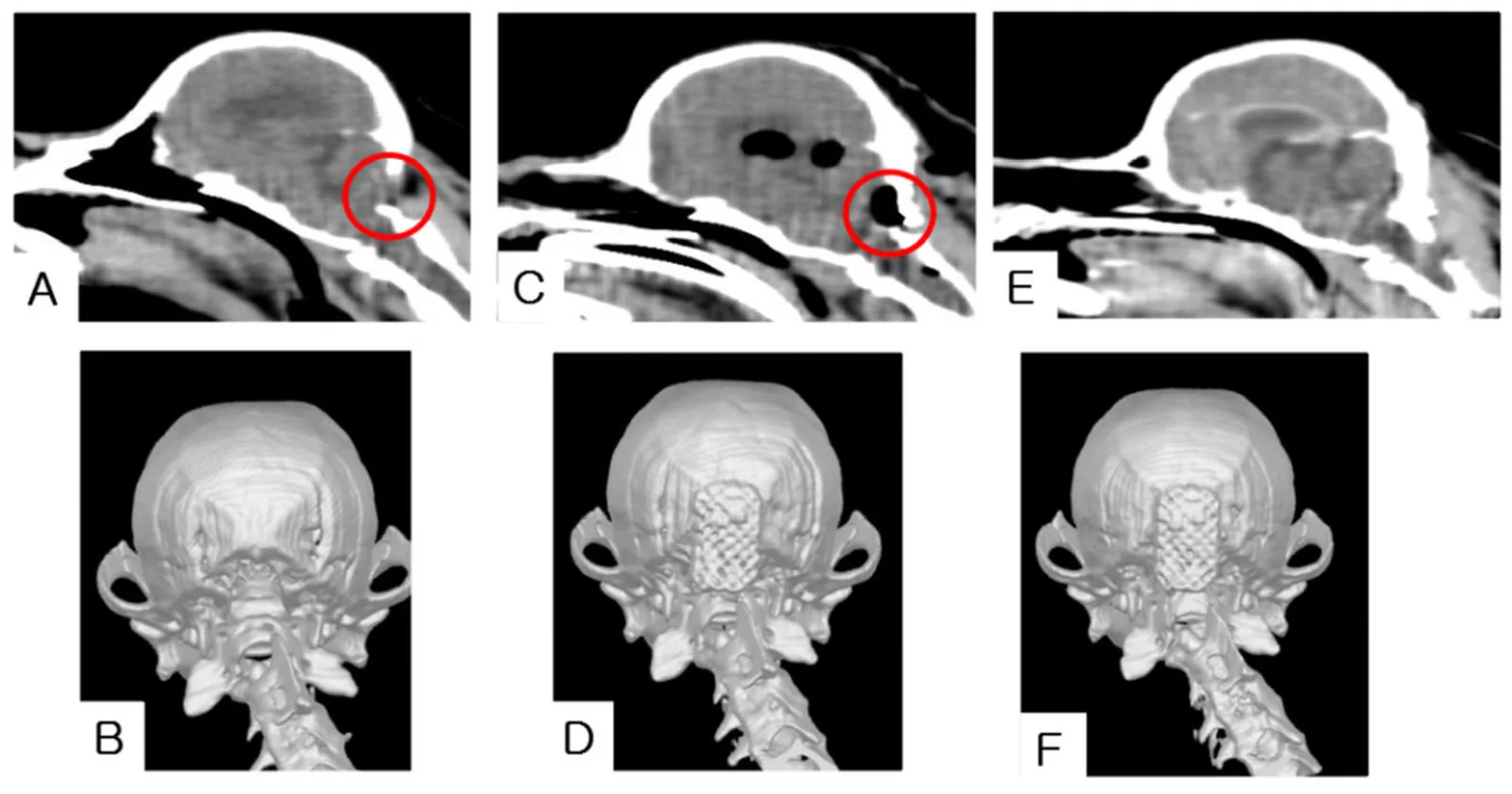
Computed tomography (CT) findings obtained before surgery, immediately after surgery, and at the 2-year postoperative follow-up following foramen magnum decompression (FMD) with titanium mesh cranioplasty. (**A**) Preoperative sagittal CT image demonstrating caudal occipital hypoplasia and dorsal overlapping of the atlas (C1) relative to the occipital bone, consistent with atlanto-occipital overlapping (AOO) (red circle). (**B**) Three-dimensional reconstructed preoperative CT image demonstrating caudal occipital bone deficiency and severe narrowing of the foramen magnum region associated with CM. (**C**) Immediate postoperative sagittal CT image obtained after FMD, C1 dorsal laminectomy, and titanium mesh plate cranioplasty. The red circle highlights the same atlanto-occipital overlapping region identified in panel (**A**), demonstrating decompression of the craniocervical junction following removal of the dorsal lamina of C1. A postoperative hypodense space containing air attenuation is visible dorsal to the cerebellum, consistent with expected postoperative intracranial air. (**D**) Three-dimensional CT reconstruction immediately after surgery demonstrating titanium mesh plate reconstruction. (**E**) Sagittal CT image obtained approximately 2 years postoperatively demonstrating maintained decompression and stable positioning of the titanium mesh implant without evidence of recurrent caudal fossa narrowing or implant displacement. Mild soft tissue formation surrounding the titanium mesh implant is observed. (**F**) Three-dimensional reconstructed CT image obtained approximately 2 years postoperatively demonstrating long-term structural stability of the titanium mesh cranioplasty and maintenance of the decompressed caudal fossa architecture.

Combined surgical decompression and immunosuppressive therapy were subsequently initiated. Preoperative CT additionally demonstrated caudal occipital hypoplasia with dorsal overlapping of the atlas (C1) relative to the occipital bone, consistent with atlanto-occipital overlapping (AOO) at the craniocervical junction (Figure 3A,B). The overlapping dorsal lamina of C1 identified within the AOO region was included in the planned decompression procedure. FMD with titanium mesh plate cranioplasty was performed using the surgical technique previously described by the authors [9], which included removal of the dorsal lamina of C1. Immediate postoperative CT confirmed adequate decompression and appropriate titanium mesh plate positioning (Figure 3C,D). Compared with the preoperative CT examination, removal of the dorsal lamina of C1 resulted in decompression of the AOO region at the craniocervical junction (Figure 3C). A postoperative hypodense region containing air attenuation was observed dorsal to the cerebellum, a finding consistent with expected postoperative intracranial air following FMD (Figure 3C). Follow-up CT performed approximately 2 years postoperatively demonstrated maintained decompression and stable implant positioning without evidence of recurrent caudal fossa narrowing or displacement, with mild perimplant soft tissue formation similar to previously reported postoperative findings (Figure 3E,F) [5].

Concurrent with surgical management, immunosuppressive therapy consisting of leflunomide (5 mg/kg, once daily) and mycophenolate mofetil (MMF; 10 mg/kg, once daily) was initiated using the protocol previously described by the authors [10]. The dosages of leflunomide and MMF were maintained without tapering throughout the follow-up period. Serial hematologic and biochemical evaluations performed approximately 6 months and 2 years after treatment initiation revealed no clinically significant adverse effects, and periodic hematologic and biochemical monitoring remained unremarkable throughout the follow-up period. Although leflunomide is frequently administered twice daily in veterinary medicine, a once-daily regimen was selected in this patient based on the author’s previous clinical experience with the same protocol [10] and concerns regarding potential cumulative adverse effects in a small-breed dog receiving combination immunosuppressive therapy. This rationale was further supported by the patient’s sustained clinical stability and unremarkable hematologic profiles throughout the extended evaluation period.

Progressive neurologic improvement was observed following combined surgical and medical treatment. Cervical discomfort gradually improved, gait abnormalities stabilized, and no further progression of neurologic dysfunction was observed during the postoperative period. Approximately 2 years after surgery and initiation of immunosuppressive therapy, the dog remained neurologically stable without recurrence or worsening of clinical signs, and no paresis, seizure activity, or additional neurologic deficits were observed during long-term follow-up. The patient maintained a normal quality of life during continued maintenance immunosuppressive therapy. Serial clinical and laboratory evaluations were performed at approximately 6 months postoperatively and annually thereafter until the final 2-year follow-up. Throughout this period, owner compliance remained excellent without treatment interruption, and no clinically significant adverse effects, including gastrointestinal signs or clinicopathologic evidence of hepatic or other organ toxicity, were observed (Table 1).

## 3. Discussion

The present report describes the long-term management of a dog with concurrent CM-associated SM and presumptive MUO. The primary clinical relevance of this case lies in the recognition of MRI lesions suggestive of an additional inflammatory intracranial disease process during repeat MRI evaluation of a patient with a previously established structural neurologic disorder, which substantially altered diagnostic interpretation and therapeutic planning.

Progressive neurologic deterioration in dogs with CM/SM is frequently attributed to persistent structural abnormalities and associated CSF flow disturbances. In the present case, however, repeat MRI demonstrated multifocal intra-axial lesions suggestive of a presumptive inflammatory meningoencephalitic process, with MUO regarded as the leading differential diagnosis. Although these lesions were not recognized on the initial MRI examination, it cannot be excluded that subtle inflammatory abnormalities may have been present but remained radiologically unapparent at that time. Regardless of their temporal development, these lesions substantially altered both diagnostic interpretation and therapeutic planning. Importantly, the extent to which the patient’s neurologic deterioration was attributable to persistent structural abnormalities, presumptive inflammatory disease, or a combination of both processes remains uncertain. It is possible that inflammatory disease and associated intracranial pathophysiologic changes contributed substantially to the observed clinical progression. This case therefore emphasizes that progressive worsening in patients with a previously established structural diagnosis should not automatically be attributed solely to the original disorder. When neurologic signs that had previously responded to medical therapy become refractory or continue to worsen, repeat MRI evaluation should be strongly considered to identify possible concurrent disease. Recent studies evaluating MRI evolution and relapse patterns in canine MUO have similarly highlighted the importance of repeat neuroimaging during disease progression and clinical reassessment [11,12].

The coexistence of CM/SM, AOO, and presumptive MUO created a therapeutic challenge because these disorders may contribute to neurologic dysfunction through distinct but potentially interacting mechanisms. Current concepts of canine Chiari-like malformation recognize the disorder as a complex developmental abnormality involving both the skull and the craniocervical junction rather than cerebellar compression alone. AOO has been described as one of several craniocervical junction abnormalities that may coexist with CM/SM and contribute to altered cerebrospinal fluid dynamics [13].

Persistent craniocervical structural abnormalities were present on both MRI examinations, whereas repeat MRI demonstrated additional multifocal intra-axial lesions suggestive of a presumptive inflammatory meningoencephalitic process, with MUO regarded as the leading differential diagnosis.

Because objective progression of the structural abnormalities could not be reliably determined and the inflammatory lesions may have been a major contributor to the clinical deterioration, the present report does not establish whether neurologic worsening was driven predominantly by structural disease, presumptive inflammatory disease, or both.

It is also possible that AOO and caudal occipital hypoplasia, rather than progression of classic CM-associated cerebellar compression alone, represented important structural contributors to caudal fossa crowding in the present case.

Nevertheless, combined treatment was elected to address both recognized disease processes. The structural component was managed surgically by FMD, C1 dorsal laminectomy, and titanium mesh plate cranioplasty to decompress the AOO region and stabilize the abnormal caudal occipital defect, whereas presumptive inflammatory disease was managed medically using long-term immunosuppressive therapy with leflunomide and MMF. The respective contribution of surgical decompression and immunosuppressive therapy to the favorable clinical outcome cannot be determined from this report. Following the initiation of combined surgical decompression and immunosuppressive therapy, the patient remained neurologically stable without recurrence or progression of clinical signs for approximately 2 years. However, because both interventions were instituted concurrently, this clinical course should not be interpreted as evidence supporting the efficacy of either treatment individually.

Several limitations should be acknowledged. This report describes only a single patient, and definitive conclusions regarding causality, treatment efficacy, or the relationship between CM/SM and MUO cannot be established. Histopathologic confirmation, cerebrospinal fluid analysis, and postoperative MRI follow-up were unavailable in the present case. Consequently, definitive confirmation of an immune-mediated inflammatory disease was not possible, and the diagnosis remained presumptive throughout the study. Therefore, the diagnosis was based on the overall integration of the clinical presentation and MRI findings, with presumptive MUO regarded as the leading differential diagnosis. Although infectious, vascular, and neoplastic diseases could not be definitively excluded, they were considered less likely based on the overall clinicoradiological assessment. This interpretation was based on the multifocal lesion distribution involving both the forebrain and brainstem, the MRI signal characteristics, the absence of imaging features strongly suggestive of a focal neoplastic process or vascular infarction, and the lack of clinical evidence supporting systemic infectious disease. Although these considerations favored a presumptive inflammatory meningoencephalitic process, definitive etiologic differentiation was not possible without cerebrospinal fluid analysis and histopathologic confirmation, as MRI characteristics alone cannot reliably distinguish inflammatory, neoplastic, and vascular intracranial diseases in dogs [14]. Postoperative MRI follow-up was not performed owing to practical and financial client constraints, representing a major limitation of this report as objective assessment of inflammatory lesion resolution or structural changes could not be obtained.

Although long-term neurological stability was observed during the approximately 2-year follow-up period, this clinical course should not be interpreted as confirmatory evidence of MUO or as evidence supporting the efficacy of either surgical decompression or immunosuppressive therapy individually, because both interventions were initiated concurrently and postoperative MRI confirmation was unavailable. The glucocorticoid-sparing regimen consisting of leflunomide and mycophenolate mofetil was selected to minimize the potential adverse effects associated with prolonged corticosteroid administration in a small-breed dog while providing complementary immunosuppressive mechanisms. This treatment strategy was also informed by our previously reported single-case experience demonstrating successful long-term remission using the same steroid-free protocol. Both drugs were initiated at once-daily dosing as a minimum effective regimen, with the intention of escalating treatment only if clinical deterioration was observed. Because the patient remained clinically stable throughout follow-up without evidence of disease progression or treatment-related adverse effects, dose escalation or tapering was not considered necessary. Additionally, evidence supporting this immunosuppressive protocol remains limited, and the rationale for selecting leflunomide and MMF in the present case was based primarily on the previously reported single-patient experience [10], which warrants cautious interpretation. Accordingly, dosing should be individualized according to each patient’s clinical response and tolerability, with careful clinical and laboratory monitoring throughout long-term therapy.

In conclusion, this report highlights the importance of repeat MRI evaluation in neurologic patients exhibiting progressive deterioration despite previous treatment, even when an established structural diagnosis is present. MRI findings suggestive of concurrent inflammatory disease should remain an important differential consideration in such patients. In this patient, clinical stability was observed following combined surgical decompression and immunosuppressive therapy. However, because both interventions were initiated concurrently, the relative contribution of each treatment remains uncertain, and no causal inference regarding treatment efficacy can be drawn from this single case. Further studies involving larger case numbers and long-term imaging follow-up are required before broader conclusions can be drawn.

## Figures and Tables

**Table 1:** Chronological summary of the clinical course, diagnostic evaluation, treatment, and long-term outcome of the present case.

Time Point	Clinical Signs	Imaging Findings	Treatment	Outcome
Approximately 12 months before referral	Episodic cervical rigidity and discomfort	CM-associated caudal fossa overcrowding, ventriculomegaly, and cervical SM on MRI	Prednisolone and acetazolamide	Temporary improvement followed by progressive neurological deterioration
Referral/preoperative reassessment	Cervical hyperesthesia, ambulatory tetraparesis, proprioceptive deficits	Persistent CM/SM with additional multifocal intra-axial MRI lesions suggestive of a presumptive inflammatory meningoencephalitic process	Repeat MRI, surgical planning	Concurrent structural and presumptive inflammatory disease considered
Surgery	Persistent neurological deficits	Pre/post operative CT confirmed caudal occipital hypoplasia and AOO	FMD, C1 dorsal laminectomy, titanium mesh cranioplasty, leflunomide + MMF	Uneventful recovery
Approximately 6 months	Clinical improvement	Follow-up CT demonstrated maintained decompression and stable titanium mesh plate position	Continued immunosuppressive therapy	Stable without adverse effects
Approximately 2 years	Neurologically stable; mild intermittent leaning gait persisted without progression	Follow-up CT demonstrated maintained decompression and stable titanium mesh plate position. Postoperative MRI was not performed.	Continued maintenance immunosuppressive therapy	Stable quality of life without disease progression

## Data Availability

The original contributions presented in this study are included in the article. Further inquiries can be directed to the corresponding author.
